# Scopolamine as a Hypnotic and Sedative in Mental Diseases

**Published:** 1906-04

**Authors:** 


### Scopolamine as a Hypnotic and Sedative in Mental Diseases.

In *These de Paris* (1905) with this title, M. Chollet reports upon the use of the scopolamine hydrobromide, hypodermically, in doses of $\frac{1}{2}$ milligram to $1\frac{1}{2}$ milligrams (gr. to ) as a hypnotic and sedative, in thirty cases, which were suffering with general paralysis and acute and chronic mania. He found that there was a rapid hypnotic effect exercised upon all cases of insomnia due to agitation. In from ten to thirty minutes after the injection, the patient went to sleep, and remained so for from five to eight hours. On the following day the patient was generally calmer, but the sedative action was always less marked than the hypnotic effect. With the exception of some vomiting, no evidence of intoxication was produced. M. Brelet, in reviewing for the *Archives generales de medicine* (January 2, 1906), points out the fact that the treatment is not free from risk. He believes that the advice should have been given with some reserve, since very serious and even fatal accidents have followed those injections of scopolamine. Therefore great prudence should be exercised in the employment of this medicament, which, moreover, has been shown by Stella to exert a toxic influence upon the respiratory centers and the myocardium.—*N. Y. Med. Jour.*

